# Revolutionizing Lyme disease vaccination: a systematic review and meta-analysis of promising candidates

**DOI:** 10.3389/fcimb.2025.1554360

**Published:** 2025-04-25

**Authors:** Sadia Tamanna, Dong-Min Kim

**Affiliations:** Department of Internal Medicine, College of Medicine, Chosun University, Gwangju, Republic of Korea

**Keywords:** Lyme disease, *Borrelia*, vaccine, recombinant protein, infection, OspA

## Abstract

The most prevalent vector-borne diseases in North America and Europe is still Lyme disease, which is caused by the bacterium *Borrelia burgdorferi*. As incidence rates rise, this poses a serious threat to public health. Since there is presently no vaccine for Lyme disease that is suitable for human use after the LYMErix vaccine was withdrawn in 2002 due to safety issues and insufficient adoption, there is an urgent need for an effective vaccination to protect at-risk populations. Numerous intriguing vaccine candidates have been developed as a result of advances in molecular biology and immunology; nevertheless, it is still unclear which candidate provides the best balance of durability, safety, and efficacy. The purpose of this meta-analysis and systematic review is to assess the safety and effectiveness of many Lyme disease vaccine candidates that are presently undergoing clinical trials. According to PRISMA guideline, the systematic review was performed, and the meta-analysis was performed using random-effect model. This study evaluates the efficacy of multiple Lyme disease vaccine candidates and identifies recombinant OspA-based formulations as the most promising by combining data from observational studies and randomized controlled trials. With an emphasis on OspA-based and multivalent vaccinations, we present comparative evaluations of immune responses, side effects, and long-term protection across vaccine platforms. This research is to help steer public health policy and vaccine development activities in the direction of a successful Lyme disease vaccine and emphasizes how certain vaccine candidates may lessen the impact of Lyme disease.

## 1 Introduction

**Figure 1:**
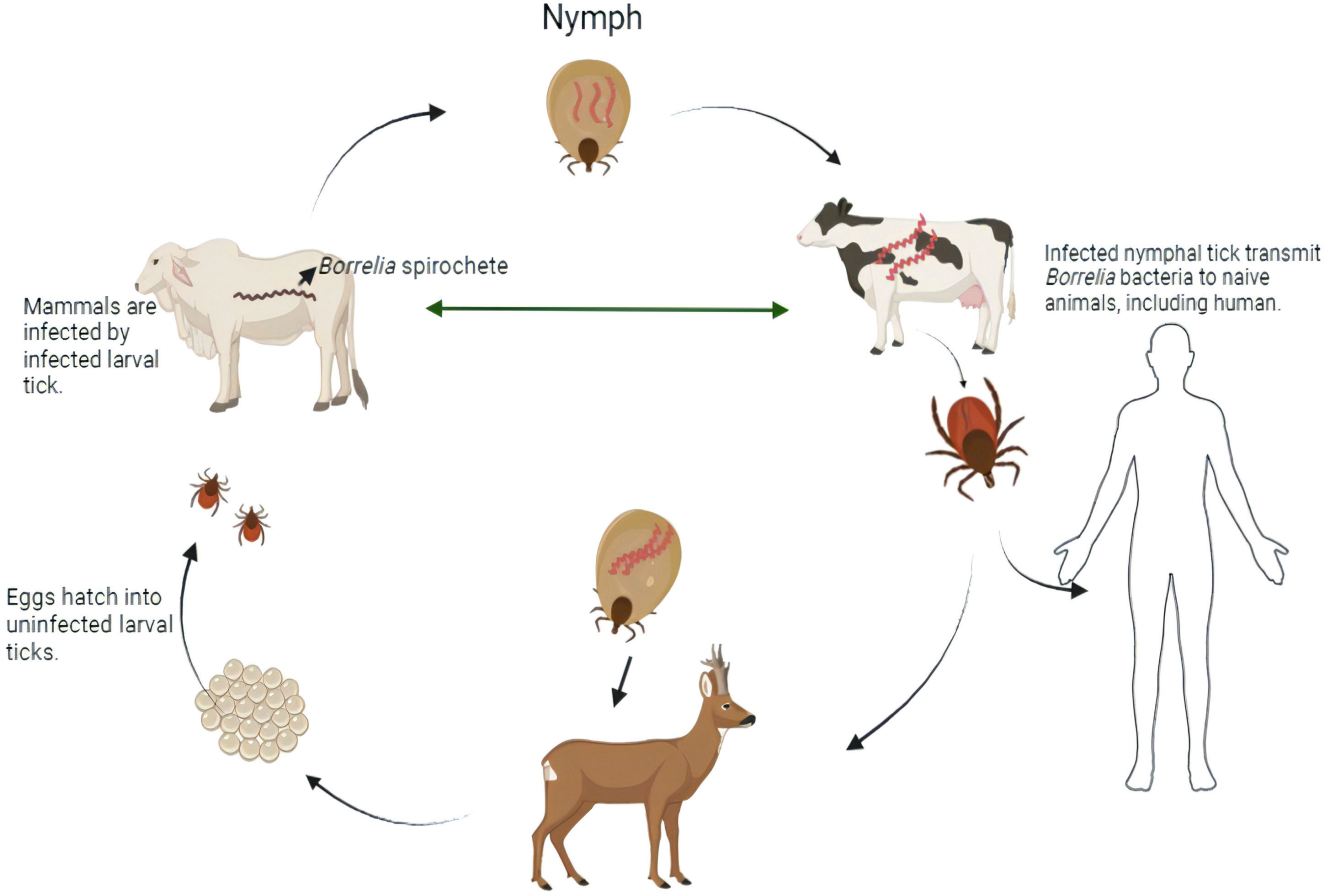
Transmission cycle of Lyme-disease-infected ticks carry the spirochetes of *Borrelia* bacteria into deer and laid eggs. From tick eggs, new nymphs are born and got infected when they get in touch with an infected mammal. Those infected nymph carry the *Borrelia* spirochetes into other naive animals including human during the time of feeding. By this, infected ticks again lay eggs into deer, and the life cycle is moved on [created in BioRender.com].

Lyme disease, commonly referred to as Lyme borreliosis, is still spreading geographically and increasing in frequency. It is difficult to eradicate the disease because of its intricate biological cycle, which includes ticks and a variety of vertebrate hosts (**Figure 1**). In the US and Asia, Lyme disease is the most well-known tick-borne disease (Steere et al., 2004). Despite the fact that this genus has at least 13 identified species, three species— *Borrelia burgdorferi* sensu stricto, *Borrelia afzelii*, and *Borrelia garini*—are mostly responsible for mammalian infection (Steere et al., 1998). While two of the three species—*B. afzelii* and *B. garini*—are widespread in Asia (Sigal et al., 1998), only *B. burgdorferi* is observed in the United States (Burgdorfer et al., 1982; Benach et al., 1983; Sigal et al., 1998; Steere et al., 1998; Steere et al., 2004; Jámbor et al., 2008; Hook et al., 2022). All three species are discovered in Europe (Sigal et al., 1998). According to a newly revealed forecast dependent on insurance documents, there are over 476,000 Americans who are infected and hospitalized for Lyme disease annually (CDC, 2021). This number represents a 59% increase over the 300,000 prediction from the former analysis (CDC, 2021).

Lyme borreliosis was identified as a significant newly emergent illness in the late twentieth century (Steere et al., 1998). A previously unnamed spirochetal bacteria known as *B. burgdorferi* was found in an *Ixodes scapularis* nymph tick by Burgdorfer and associates in 1981 (Burgdorfer et al., 1982; Hook et al., 2022). After that, spirochetes were grown from individuals who had early-stage Lyme disease, and a clear connection was found between the microbe and the patients’ immunological responses, indicating the spirochetal origin of the condition (Benach et al., 1983; Steere et al., 1983).

From early symptoms like flu-like symptoms and erythema migrans, a distinctive bullseye rash, to later-stage consequences affecting the joints, neurological system, and cardiovascular system, Lyme disease can present with a wide range of symptoms. Affected people’s quality of life may be severely impacted if the condition is left untreated because it can result in long-lasting health problems like Lyme arthritis and neuroborreliosis.

The exploration of a Lyme disease vaccine began in the 1980s when researchers identified the causative agent and understood its transmission through tick bites (Wormser et al., 2000). In the late 1980s and early 1990s, the first-generation Lyme disease vaccine (Barone et al., 2002; Cheff, 2015), LYMErix, emerged as a promising candidate. Developed by SmithKline Beecham (now GlaxoSmithKline), LYMErix utilized a protein called OspA, a surface protein of the *B. burgdorferi* bacterium (Barone et al., 2002). This OspA vaccine shared arthritogenic T-cell epitope (OspA aa165–173) mimicking human leukocyte function-associated antigen-1 that induced some potential side effects, including reports of arthritis-like symptoms (Halsey et al., 2000). Therefore, the company voluntarily withdrew LYMErix from the market in 2002 (Halsey et al., 2000; Hitt, 2002). Since then, there has been no approved vaccine for human usage, creating a serious weakness in the defenses against Lyme disease.

The development of a vaccine has been a key priority in response to the rising incidence of Lyme disease. Recent developments in immunology, however, have rekindled interest in vaccination research (Wormser et al., 2000; Hitt, 2002; Plotkin, 2011). Preclinical and clinical trials have shown promise for a number of investigational vaccines, including multivalent and outer surface protein A (OspA)-based options (Fikrig et al., 1992; Zumstein et al., 1992; Wressnigg et al., 2013).

Thus, it is imperative to assess the present state of vaccination candidates for Lyme disease. To analyze the efficacy and safety profiles of candidates, a thorough synthesis of the available data is required, even though individual studies offer insightful information. To date, no other research has examined the effectiveness of Lyme vaccine candidates out of all those under investigation. Furthermore, there are still unanswered questions about these vaccines’ long-term protection and population-specific efficacy. By methodically examining the data that are now available, contrasting potential vaccines, and determining which are the most effective and practical choices for use in the future, this review seeks to close these gaps. By taking this strategy, the purpose of this study is to shed more light on how vaccinations for Lyme disease can lessen the disease’s incidence and impact.

## 2 Methods

### 2.1 Search strategy

A comprehensive literature search was conducted across PubMed/MEDLINE, Google Scholar, Web of Science, and the Cochrane Library, covering all relevant publications up to 10 October 2024. The search focused on randomized controlled trials evaluating potential vaccine candidates for Lyme disease. Key search terms included “Lyme disease vaccine,” “*Borrelia* infection,” “potential vaccine candidates for Lyme,” “OspA-based vaccines,” “OspC-based vaccine,” “Salp15 antigen,” “Salp25 antigen,” “successful vaccine candidates,” and “vaccine antigens.” In PubMed, specific Medical Subject Headings (MeSH) terms were also incorporated to refine and enhance the search strategy.

### 2.2 Study selection process

**Figure 2:**
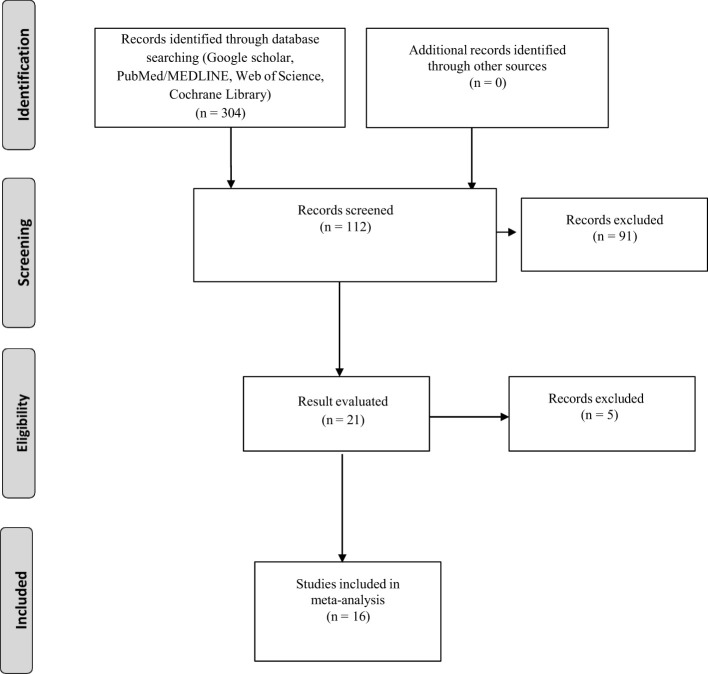
Process of systematic review for this study.

After the database searches, EndNote X7 (Thomson Reuters, Toronto, ON, Canada) was used to manage duplications and combine the records that were gathered. Each record was initially screened by reviewing the titles and abstracts to exclude studies that did not align with the study’s objectives. Excluded from this pool were studies with no experimental data. Studies without a data table displaying the number of infected and protected hosts after immunization were also disqualified. Lastly, research that lacked a control group was disqualified from consideration. The remaining research that satisfied every requirement was chosen to be part of the meta-analysis (**Figure 2**).

### 2.3 Data extraction

Data files that were stored in a Microsoft Excel spreadsheet (XP Professional Edition; Microsoft Corp, Redmond, WA) were used for the data extraction method. Important details including the first author’s name, the year of publication, the study design, the definitions of the test and control groups, the sample sizes for both groups, and the study results were among the information that was retrieved.

### 2.4 Meta-analysis

Random-effects model was applied in this study, which is more conservative and appropriate for heterogeneity studies than other model. Cochran’s Q, I^2^, τ², and H^2^ statistics were performed to determine the significant heterogeneity of this study. The Wald test was used to determine Fisher’s z value, and the combined results were shown as estimates. The confidence interval (CI) at the 95% confidence level were computed to determine the meta-analysis effect size. To determine the relationship between each variety, the Pearson correlation coefficient from the research was also computed. File-drawer analysis was used to investigate potential publishing bias; a greater fail-safe N denotes the relevance of this analysis. Comprehensive Meta-Analysis Software, Version 3.0 (Biostat, Englewood, NJ), was used for all analytical processes.

## 3 Results

From an initial pool of 304 articles, 16 specific studies were identified that met the inclusion criteria on this study (**Figure 2**). Estimates of analyzed variables are shown in **Table 1**, along with their corresponding 95% confidence intervals (CIs). Recombinant OspA, BB0172-derived peptide, BbHtrA, BB0689, RevA, recombinant P66, recombinant VlsE, lipidated recombinant protein, CspZ, and protein with nanoparticles (such as ferritin, LNP) are used as vaccine candidate among included studies that showed potential efficacy against Lyme bacteria (**Table 2**).

**Table 1:** Coefficients of this study.

					95% Confidence Interval	
	Estimate	Standard Error	z	p	Lower	Upper
intercept	0.870	0.210	4.139	<0.001	0.458	1.283
tpos	0.005	0.003	1.718	0.086	-6.821×10^-4^	0.010
tneg	0.016	0.005	3.399	<0.001	0.007	0.025
cpos	-0.037	0.011	-3.369	<0.001	-0.059	-0.016
cneg	-0.044	0.100	-0.440	0.660	-0.240	0.152

Wald test.

**Table 2:** Characteristics of included studies, animal models, and vaccine candidates.

Study characteristics				Animal characteristics			Characteristics of vaccine candidates		
Year	First author	Country	Study period	Model	Age	Gender	Antigen	Route	Dose (per dose)
1996	de Silva	USA	–	C3H/HeN mice	–	–	OspA-GT fusion protein (rabbit antiserum)	Intraperitoneal, Subcutaneous	200 μl antiserum
1997	Gern	Switzerland	15 weeks	BALB/c	–	Female	Lipidated, recombinant OspA [from strains ZQI (*B.* *garinii*) and ACAl (*B. afzelii*)]	Subcutaneous	1 μg, 0.1 μg
1999	Cindy	USA	6 weeks	LSH Hamster	12–16 weeks	–	rOspA	Intramuscularly	120 μg, 60 μg, 30 μg
2011	Livey	USA	8 weeks	C3H/HeJ mice	–	–	rOspA ½ (The proximal portion of serotype-1 OspA sequence (*B. burgdorferi*) fused to the distal portion of serotype-2 OspA (*B. afzelii*)	Subcutaneous	0.1 μg, 0.03 μg
2014	Small	USA	16 weeks	C3H/HeN mice	6 weeks	Female	BB0172-Derived Peptide	Intramuscularly	50 μg
2015	Ullmann	USA	16 weeks	C3H/HeJ mice	5–6 weeks	Female	BbHtrA	–	18 μg
2015	Byram	USA	4 weeks	C3H/HeN mice	4–6 weeks	Female	r RevA introduced into *B. burgdorferi* B31	Subcutaneous	1×10^5^, 1×10^4^, 1×10^3^, 1×10^2^ of bacteria
2015	Comstedt	Austria	15 weeks	C3H/HeN mice	8 weeks	Female	OspA (Lip-D2B1B-His, Lip-M1B-His)	Subcutaneous	5 μg, 2 μg, 2.5 μg
2016	Hahn	USA	33 weeks	C3H/HeN mice	3 weeks	Female	rP66 (Deletion of p66 in B31-A3, overproduces P66)	Subcutaneous	1×10^5 cells^ in 0.1 ml
2018	Marcinkiewicz	USA	8 weeks	C3H/HeN mice and Swiss Webster mice	3 weeks	Male	CspZ- VLP	Intraperitoneal	25 μg
2020	Klouwens	Netherlands	≈ 9 weeks	C3H/HeN mice	6–8 weeks	Female	rOspA+ meningococcal Outer Membrane Vesicles	Subcutaneous	40 μg, 4.2 μg
2020	Kamp	USA	8 weeks	C3H/HeN mice, Rhesus monkey		Male & Female	OspA-GS-ferritin	Intramuscularly	Mice (1 and 6 μg), monkey (60 μg)
2020	Nayak	Austria	15 weeks	C3H/HenRj mice	8–10 weeks	Female	chimeric OspA	Subcutaneous	5 μg
2022	Batool	USA	6 weeks	C3H/HeN mice	4–6 weeks	Male	host-adapted rVlsE	Subcutaneously transplantation	1.5-mm diameter
2023	Šaško	USA	16 weeks	C3H/HeNHsd Mice, BALB/ cOlaHsd mice	5 weeks, 7 weeks	Female	QVLP-BB0689	Subcutaneous	25 μg
2023	Pine	USA	24 weeks	Balb/c	8 weeks	Female	OspA mRNA-LNP	Intramuscularly	3 μg

After infection of vaccinated mice and hamster model, the infected host group is indicated by test positive (t-pos), and the protected host group is indicated as test negative (t-neg). On the other hand, the infected host in the control group is indicated by control positive (c-pos), and the non-infected host in the control group is indicated by control negative (c-neg). All of these studies were randomized trials with either mouse model or hamster model.

### 3.1 Animal model

When assessing the safety and effectiveness of Lyme vaccinations, animal models are crucial. Because of their reliable and strong immune system, BALB/c mice were widely utilized to investigate immune responses in the early phases of Lyme disease vaccine research (Golde et al., 1995; Zhong et al., 1997; Kumar et al., 2011). Important information about antibody production and general vaccine-induced protection was obtained from these mice. The BALB/c model’s incapacity to accurately depict Lyme-disease-associated arthritis, a prevalent and dangerous clinical consequence, is a major drawback, though (Barthold et al., 1990; Seiler et al., 1995). The need for more thorough models that could more accurately replicate real disease was brought to light by the launch of the LYMErix vaccine, which was taken off the market when complaints of side effects surfaced. To properly evaluate immunological responses and the possibility of joint inflammation after immunization, researchers resorted to the C3H/HeN mouse model, which is more prone to *B. burgdorferi*-induced arthritis (Fikrig et al., 1990; Nardelli et al., 2010; Marcinkiewicz et al., 2018). Due to its ability to replicate the entire range of Lyme pathology, including the onset of arthritis, which is a crucial component in assessing vaccine safety, the C3H mouse model has since been employed in Lyme disease vaccine studies (Fikrig et al., 1990; Marcinkiewicz et al., 2018). For Lyme vaccination experiments, scientists have also used Guinea pigs and Syrian hamsters in addition to mice (Sonnesyn et al., 1993; Lovrich et al., 2005). These have the benefit of exhibiting more severe infection signs, such as carditis and arthritis, which makes them an excellent model for researching the protective effects of immunization and the pathogenic effects of *Borrelia*. However, because of real-world constraints like size and availability, they are not as often employed as mouse models.

### 3.2 Construction of vaccine candidates

Most of the vaccine candidates used basically OspA protein of *Borrelia* bacteria as antigen included in this study. However, OspA is not inserted directly in any of studies due to the limitation of previously commercialized Lyme vaccine, LYMErix. Some researchers used OspA at different dose with different adjuvants to check the level of adverse effect (Croke et al., 2000). On the other hand, some researchers inserted OspA in meningococcal outer membrane vesicles, to verify the protective effect in multiple vaccine compositions (Klouwens et al., 2021). Some researchers used serotype-1 against *B. burgdorferi* fused to the distal portion of serotype-2 from *B. afzelii* as vaccine candidate to check the protection against two species of *Borrelia* (Livey et al., 2011). Furthermore, OspA antiserum was injected with glutathione transferase (GT), and this study revealed that *Borrelia* spirochetes were transferred to salivary glands from the gut of ticks. As a result, mice were not infected, as there was no spirochetes in the tick gut that play the transmission role of Lyme infection (de Silva et al., 1996). On the other hand, recombinant *B. burgdorferi* OspA was infused with liposomes containing cobalt porphyrin-phospholipid adjuvant (Federizon et al., 2020). Liposomes enhanced the delivery of antigens to immune cells where cobalt porphyrin stimulated Toll-like receptors (TLRs) and enhanced antigen presentation by dendritic cells, and the combination of liposomes with cobalt porphyrins enhanced antigen presentation by dendritic cells. Although this candidate revealed longer protection, the durability of protection in other species is not clear (Federizon et al., 2020). Lipidated OspA was produced from two *Borrelia* species, used by some researchers as vaccine candidate to block the bacteria transmission and protect host model (Gern et al., 1997). In most of the cases, alum was used as adjuvant that triggers the release of chemokines and promote immune response according to the antigens (Zhao et al., 2023). Recently, nanoparticles like ferritin are conjugated with outer surface proteins to create a multivalent vaccine (Kamp et al., 2020). By duplicating pathogen-associated structural motifs, ferritin nanoparticles naturally generate virus-like structures that effectively boost the immune system and produce long-lasting antibody responses (Kamp et al., 2020). Additionally, researchers have developed mRNA-based Lyme disease vaccines that use lipid nanoparticles (LNPs) for effective delivery, motivated by the success of mRNA vaccines for severe acute respiratory syndrome coronavirus 2 (SARS-CoV-2) (Pine et al., 2023). Unlike protein-based vaccines, the LNP formulation prevents mRNA from degrading, enabling longer antigen presentation and a more robust immune response (Pine et al., 2023). Mice and non-human primates immunized with OspA mRNA-LNP showed elevated levels of neutralizing antibodies in preclinical investigations (Pine et al., 2023). However, some researchers also used different targets as vaccine candidate, such as BB0172, BBA52, BbHtrA, RevA, BB0689, rP66, rVlsE, and CspZ-VLP, which revealed partial protection against *Borrelia* species (Small et al., 2014; Byram et al., 2015; Comstedt et al., 2015; Hahn et al., 2016; Hassan et al., 2019; Liekniņa et al., 2023).

## 4 Discussions

The outcome of this study contribute to continued efforts to create a workable vaccine against this increasingly common infection by offering important insights into the safety and effectiveness of different Lyme disease vaccine candidates. A vaccine’s ability to be successful is multifaceted and relies on the strength, duration, and efficiency of the evoked adaptive immune responses (Lawrence et al., 2001; Del Giudice et al., 2017; Nayak et al., 2020). Conversely, adjuvants, which are employed to lessen the dosage of the vaccines and to enhance or elicit particular immune responses, are also crucial to the creation of vaccines (Vogel, 2000; De Gregorio et al., 2013; Bergmann-Leitner and Leitner, 2014). It is found that OspC and OspA proteins are not the only way that *B. burgdorferi* infection develops in the environment (Fikrig et al., 1992; Zumstein et al., 1992; Gilmore et al., 1996; Hofmeister et al., 1999). Therefore, researchers nowadays target different peptide or protein regions of *Borrelia* bacteria as vaccine target by using various methods, such as using different nanoparticles and lipoprotein to give more specific outcome by mimicking bacteria structure or method of infection (Small et al., 2014; Byram et al., 2015; Comstedt et al., 2015; Hahn et al., 2016; Hassan et al., 2019; Liekniņa et al., 2023).

In this study, the intercept (0.964) is highly significant (*p* < 0.001; 95% CI, 0.868–1.060), which indicates that the model has a meaningful baseline level and how the independent variables influence the effect size of this study. The significant Cochran’s Q value indicates that all the included studies do not share similar effect size (*p* <0.001). The main conclusions highlight the variations in vaccine-induced immunity among species and demonstrate the potential of a number of recombinant proteins, including OspA-based vaccines and other antigenic targets, to elicit protective immune responses in animal models.

The discovery of OspA-based vaccines as top contenders as a result of their proven capacity to produce notable protective immunity in both mouse and hamster models is one of the primary findings of the meta-analysis. This result is in line with LYMErix’s historical evolution, which also depended on OspA as its primary antigen. Current OspA formulations have been altered by using vesicles or nanoparticles to mimick the bacterial structure or the route of infection to get more significant outcome and to lessen the negative effects of LYMErix, including reports of symptoms arthritis.

**Figure 3:**
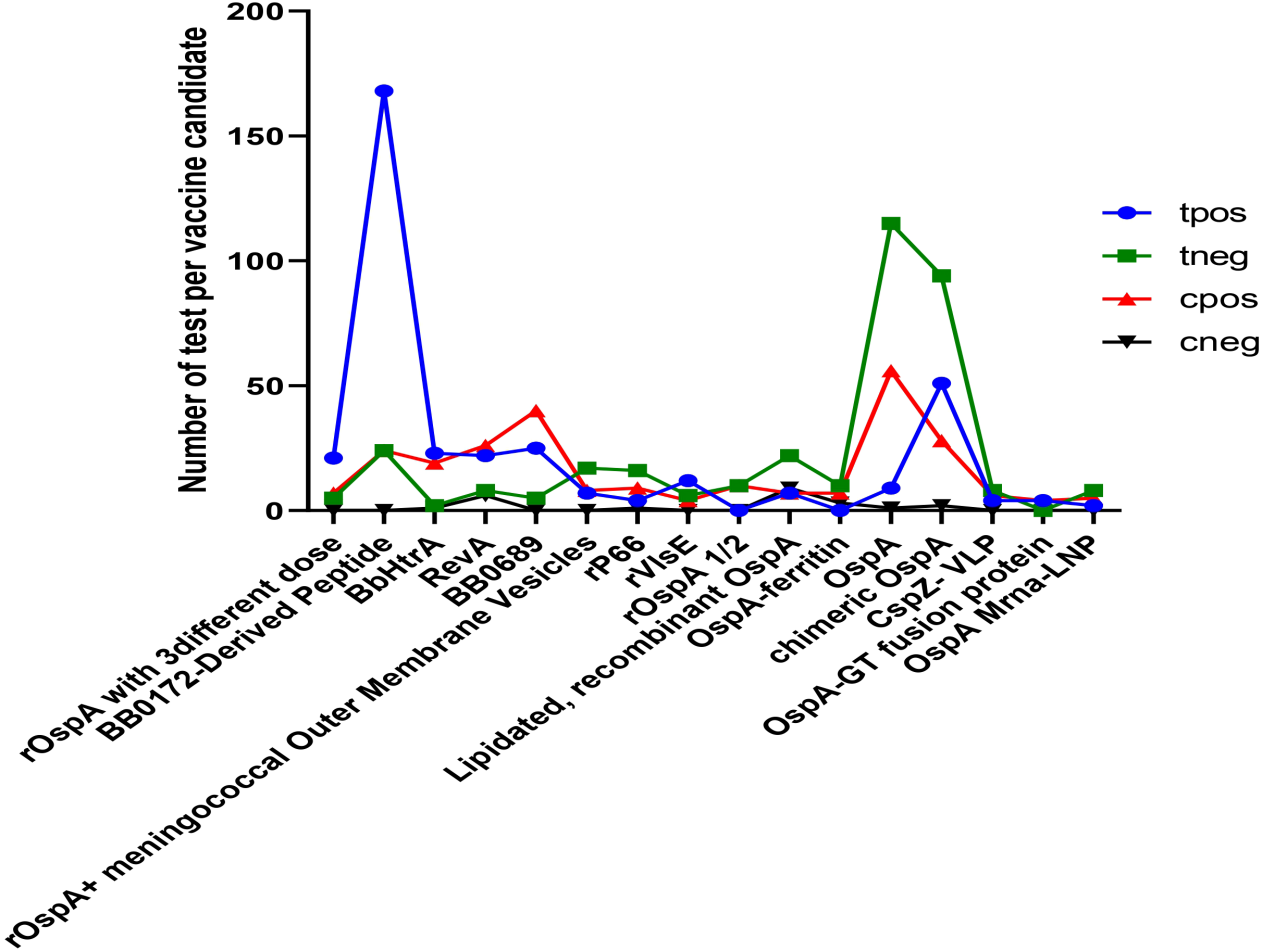
Comparison of potential vaccine candidates along with the control group. tpos indicates number of positive infection in the experimental group; tneg indicates negative infection in the experimental group; cpos indicates positive infection in the control group; and cneg indicates negative infection in control group.

**Figure 4:**
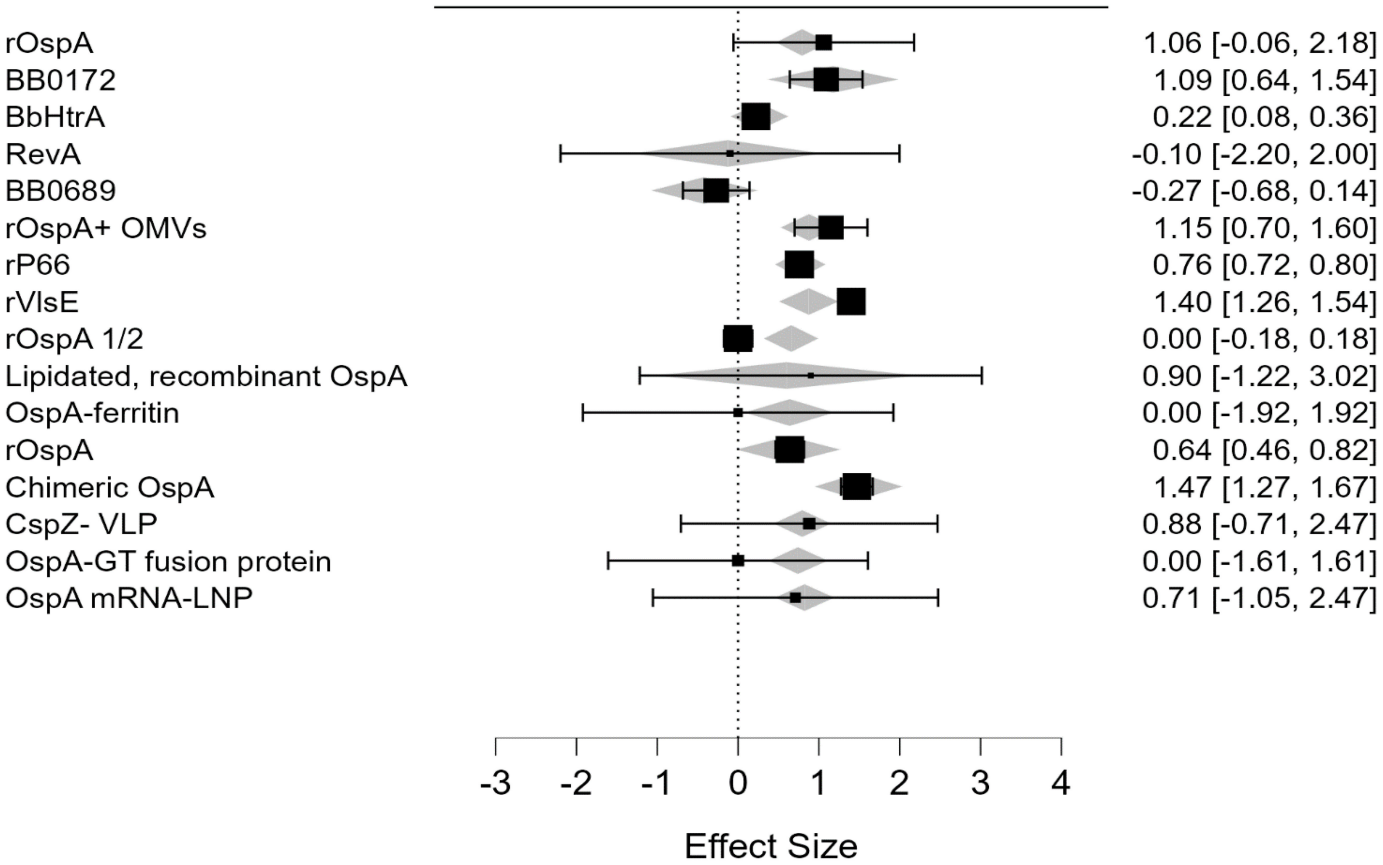
Forest plot of included studies.

Several treatments show significant positive effects, particularly BB0172-derived peptide, BbHtrA, recombinant OspA, recombinant OspA along with meningococcal outer membrane vesicles, recombinant P66, recombinant VlsE, and chimeric OspA (**Figure 3**). Among all of the analyzed vaccine candidates in this study, BB0172-derived peptide, recombinant OspA along with meningococcal outer membrane vesicles, recombinant P66, recombinant VISE, and chimeric OspA revealed stronger positive effect (**Figure 4**). On the other hand, some treatments, like RevA, BB0689, rOspA ½, OspA-Ferritin, lipidated rOspA, CspZ-VLP, and OspA mRNA-LMP, did not show any significant effects. In addition, the effectiveness is uncertain whenever recombinant OspA were used in three different doses, although it showed positive singnificant outcome.

However, using several animal models, specifically, BALB/c and C3H/HeN mice, was essential in determining the effectiveness and adverse effects of the vaccination. Although BALB/c mice’s strong immune responses made them valuable for early vaccine research, the switch to C3H/HeN mice was required since this model was unable to reproduce Lyme arthritis, a major hallmark of human Lyme disease. These mice are a more appropriate model to assess the possible adverse effects of vaccines, especially with regard to joint inflammation, because they are more prone to arthritis caused by *B. burgdorferi*. A more thorough evaluation of vaccination safety was made possible by the C3H/HeN model, which was successful in determining both the risk of arthritis and the protective advantages of vaccine candidates.

**Figure 5:**
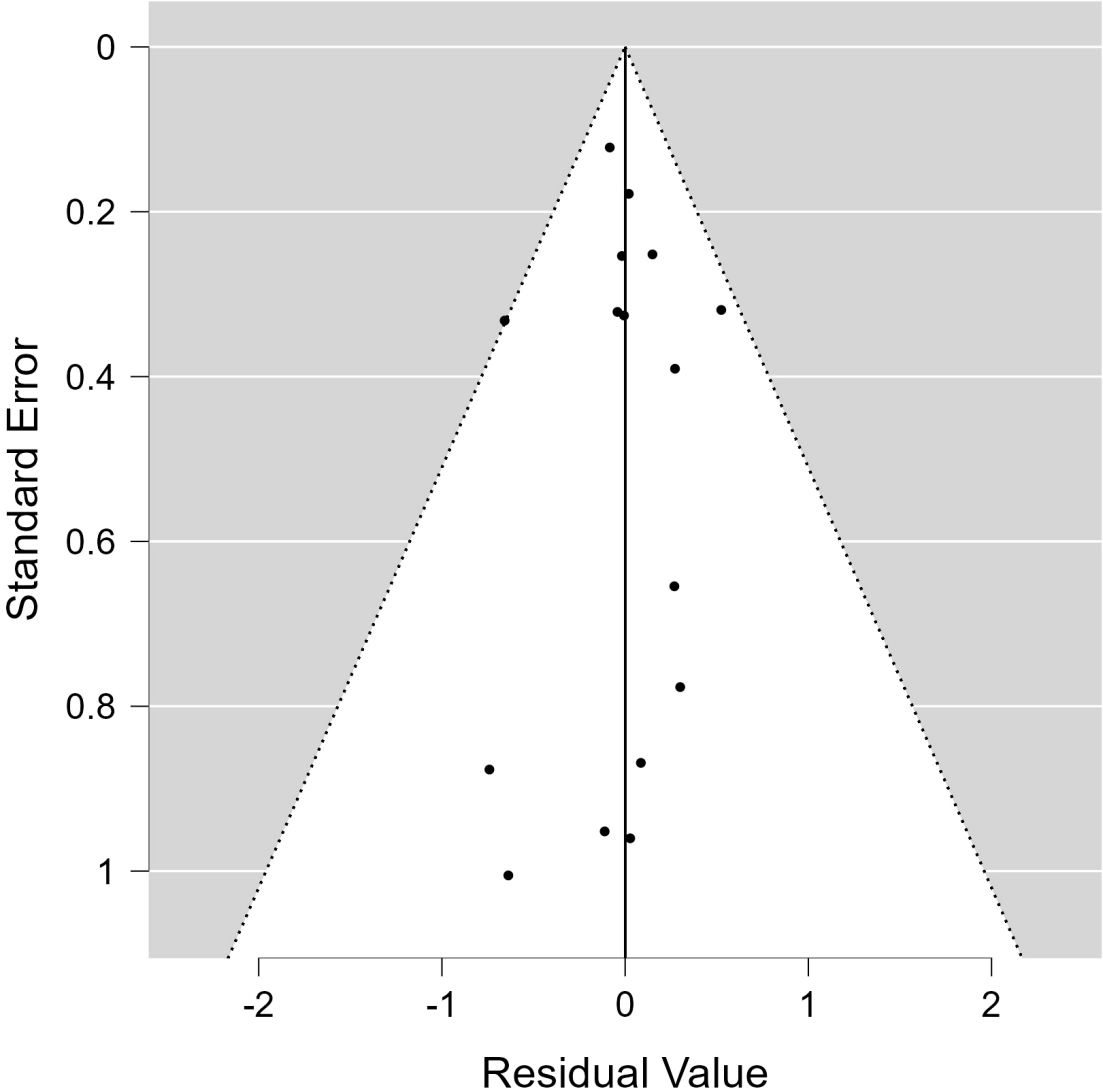
Funnel plot of included studies.

All of the predictors (tpos, tneg, cpos, and cneg) have statistically significant effects on the dependent variable, according to statistical analysis (**Table 3**). Strong connections between the infection in control groups (c-pos) and the number of protected hosts in the vaccinated group (t-neg) were found in the meta-analysis, indicating a noteworthy overall efficacy of the vaccine candidates examined. Consequently, a positive correlation with the dependent variable is suggested by positive coefficients (tpos and tneg). Furthermore, a highly significant connection between cpos and tneg is revealed by Pearson’s partial correlations (Pearson’s r = 0.802***), indicating that when one variable rises, the other tends to rise as well. The Cochran’s Q statistics, I^2^, and H^2^ showed that heterogeneity existed between studies, indicating that variations in vaccination formulations, doses, and host models might be responsible for the variations in trial efficacy (**Tables 4**, **5**). In order to guarantee more comparable data, this variation emphasizes the necessity of uniform techniques in subsequent vaccine trials. The high fail-safe N (3,382.00) in the robustness test (**Figure 5**) indicates that the results are reliable and that many unpublished research would be needed to disprove their relevance.

**Table 3:** Pearson’s partial correlations among four variables.

Variable		tpos	tneg	cpos	cneg
1. tpos	Pearson’s r	–			
	p-value	–			
2. tneg	Pearson’s r	0.003	–		
	p-value	0.992	–		
3. cpos	Pearson’s r	0.303	0.802***	–	
	p-value	0.293	<.001	–	
4. cneg	Pearson’s r	0.004	0.334	0.313	–
	p-value	0.988	0.243	0.276	–

Conditioned on variables: ES, SE.

***p <0.001.

**Table 4:** Random effects of included studies.

	Q	df	*p*
Omnibus test of model coefficients	15.275	4	.004
Test of residual heterogeneity	107.202	11	<.001

Q, Cochran’s Q statistic; df, degree of freedom; *p*, *p*-value.

The model was estimated using a random-effect method.

**Table 5:** Residual heterogeneity estimates.

		95% Confidence interval	
	Estimate	Lower	Upper
τ²	0.131	0.030	0.287
Τ	0.362	0.174	0.536
I² (%)	88.266	63.399	94.276
H²	8.522	2.732	17.471

τ², study variance; I², proportion of total variance; H², heterogeneity ratio.

## 5 Conclusion

As evidenced by the low *p*-values and confidence intervals that exclude zero, the results show a statistically significant model with many predictors influencing the outcome variables (**Table 6**). This implies that the dependent variable under study is probably going to be significantly impacted by the predictors. The results of this research highlight how vaccines based on OspA and other recombinant proteins, like p66 and VlsE, may offer protection against Lyme disease. Besides that, ampliflication the OMV-based vaccine technology (meningococcal outer membrane vesicles) along with recombiant OspA protein also has significant protection effect against Lyme borreliosis. The evidence favors the further development and testing of these vaccine candidates, even though more study is required to enhance formulations and guarantee long-term safety, especially in connection to arthritis. Future Lyme disease vaccinations must be tested for safety and efficacy using suitable animal models, such as C3H/HeN mice.

**Table 6:** File drawer analysis.

	Fail-safe N	Target Significance	Observed Significance
Rosenthal	3,382.000	0.050	<0.001

## References

[B1] Barone S. R. Bohrer S. S. Erhardt W. A. (2002). Parental knowledge of and attitudes toward LYMErix (Recombinant osp A lyme vaccine). Clin. Pediatr. 41, 33–36. doi: 10.1177/000992280204100107 11866363

[B2] Barthold S. W. Beck D. S. Hansen G. M. Terwilliger G. A. Moody K. D. (1990). Lyme borreliosis in selected strains and ages of laboratory mice. J. Infect. Dis. 162, 133–138. doi: 10.1093/infdis/162.1.133 2141344

[B3] Benach J. L. Bosler E. M. Hanrahan J. P. Coleman J. L. Habicht G. S. Bast T. F.. (1983). Spirochetes isolated from the blood of two patients with Lyme disease. New Engl. J. Med. 308, 740–742. doi: 10.1056/NEJM198303313081302 6828119

[B4] Bergmann-Leitner E. S. Leitner W. W. (2014). Adjuvants in the driver’s seat: how magnitude, type, fine specificity and longevity of immune responses are driven by distinct classes of immune potentiators. Vaccines 2, 252–296. doi: 10.3390/vaccines2020252 26344620 PMC4494256

[B5] Burgdorfer W. Barbour A. G. Hayes S. F. Benach J. L. Grunwaldt E. Davis J. P. (1982). Lyme disease—a tick-borne spirochetosis? Science 216, 1317–1319. doi: 10.1126/science.7043737 7043737

[B6] Byram R. Gaultney R. A. Floden A. M. Hellekson C. Stone B. L. Bowman A.. (2015). Borrelia burgdorferi RevA significantly affects pathogenicity and host response in the mouse model of Lyme disease. Infection Immun. 83, 3675–3683. doi: 10.1128/IAI.00530-15 PMC4534657 26150536

[B7] CDC (2021). How many people get Lyme disease? Available online at: https://www.cdc.gov/lyme/stats/humancases.html (Accessed 05 December, 2023).

[B8] Cheff B. (2015). The role of dksa in the stringent response in the lyme disease spirochete borrelia burgdorferi.

[B9] Comstedt P. Hanner M. Schüler W. Meinke A. Schlegl R. Lundberg U. (2015). Characterization and optimization of a novel vaccine for protection against Lyme borreliosis. Vaccine 33, 5982–5988. doi: 10.1016/j.vaccine.2015.07.095 26277070

[B10] Croke C. L. Munson E. L. Lovrich S. D. Christopherson J. A. Remington M. C. England D. M.. (2000). Occurrence of severe destructive Lyme arthritis in hamsters vaccinated with outer surface protein A and challenged with Borrelia burgdorferi. Infection Immun. 68, 658–663. doi: 10.1128/IAI.68.2.658-663.2000 PMC97189 10639430

[B11] De Gregorio E. Caproni E. Ulmer J. B. (2013). Vaccine adjuvants: mode of action. Front. Immunol. 4, 214. doi: 10.3389/fimmu.2013.00214 23914187 PMC3728558

[B12] Del Giudice G. Goronzy J. J. Grubeck-Loebenstein B. Lambert P. H. Mrkvan T. Stoddard J. J.. (2017). Fighting against a protean enemy: immunosenescence, vaccines, and healthy aging. NPJ Aging Mech. Dis. 4, 1. doi: 10.1038/s41514-017-0020-0 29285399 PMC5740164

[B13] de Silva A. M. Telford S. R. 3rd Brunet L. R. Barthold S. W. Fikrig E. (1996). Borrelia burgdorferi OspA is an arthropod-specific transmission-blocking Lyme disease vaccine. J. Exp. Med. 183, 271–275. doi: 10.1084/jem.183.1.271 8551231 PMC2192397

[B14] Federizon J. Frye A. Huang W. C. Hart T. M. He X. Beltran C.. (2020). Immunogenicity of the Lyme disease antigen OspA, particleized by cobalt porphyrin-phospholipid liposomes. Vaccine 38, 942–950. doi: 10.1016/j.vaccine.2019.10.073 31727504 PMC6980772

[B15] Fikrig E. Barthold S. W. Kantor F. S. Flavell R. A. (1990). Protection of mice against the Lyme disease agent by immunizing with recombinant OspA. Science 250, 553–556. doi: 10.1126/science.2237407 2237407

[B16] Fikrig E. R. O. L. Barthold S. W. Kantor F. S. Flavell R. A. (1992). Long-term protection of mice from Lyme disease by vaccination with OspA. Infection Immun. 60, 773–777. doi: 10.1128/iai.60.3.773-777.1992 PMC257553 1541551

[B17] Gern L. Hu C. M. Voet P. Hauser P. Lobet Y. (1997). Immunization with a polyvalent OspA vaccine protects mice against Ixodes ricinus tick bites infected by Borrelia burgdorferi ss, Borrelia garinii and Borrelia afzelii. Vaccine 15, 1551–1557. doi: 10.1016/S0264-410X(97)00066-2 9330467

[B18] Gilmore R. D. Jr. Kappel K. J. Dolan M. C. Burkot T. R. Johnson B. J. (1996). Outer surface protein C (OspC), but not P39, is a protective immunogen against a tick-transmitted Borrelia burgdorferi challenge: evidence for a conformational protective epitope in OspC. Infection Immun. 64, 2234–2239. doi: 10.1128/iai.64.6.2234-2239.1996 PMC174061 8675332

[B19] Golde W. T. Burkot T. R. Piesman J. Dolan M. C. Capiau C. Hauser P.. (1995). The Lyme disease vaccine candidate outer surface protein A (OspA) in a formulation compatible with human use protects mice against natural tick transmission of B. burgdorferi. Vaccine 13, 435–441. doi: 10.1016/0264-410X(94)00027-K 7639011

[B20] Hahn B. L. Padmore L. J. Ristow L. C. Curtis M. W. Coburn J. (2016). Live attenuated Borrelia burgdorferi targeted mutants in an infectious strain background protect mice from challenge infection. Clin. Vaccine Immunol. 23, 725–731. doi: 10.1128/CVI.00302-16 27335385 PMC4979176

[B21] Halsey N. A. Sikand V. K. Van Hoecke C. Buscarino C. J. Parenti D. L. (2000). “Safety and immunogenicity of LYMErix (R), Lyme disease vaccine (Recombinant OspA) in children 4 to 18 years,” in Pediatric Research, vol. 47. (Int Pediatric Research Foundation, Inc, 351 West Camden St, Baltimore, Md 21201-2436 USA), 263A–263A.

[B22] Hassan W. S. Giaretta P. R. Rech R. Ollivault-Shiflett M. Esteve-Gasent M. D. (2019). Enhanced protective efficacy of Borrelia burgdorferi BB0172 derived-peptide based vaccine to control Lyme disease. Vaccine 37, 5596–5606. doi: 10.1016/j.vaccine.2019.07.092 31387750

[B23] Hitt E. (2002). Poor sales trigger vaccine withdrawal. Nat. Med. 8, 311–313. doi: 10.1038/nm0402-311b 11927918

[B24] Hofmeister E. K. Glass G. E. Childs J. E. Persing D. H. (1999). Population dynamics of a naturally occurring heterogeneous mixture of Borrelia burgdorferi clones. Infection Immun. 67, 5709–5716. doi: 10.1128/IAI.67.11.5709-5716.1999 PMC96945 10531219

[B25] Hook S. A. Hansen A. P. Niesobecki S. A. Meek J. I. Bjork J. K. Kough E. M.. (2022). Evaluating public acceptability of a potential Lyme disease vaccine using a population-based, cross-sectional survey in high incidence areas of the United States. Vaccine 40(2) pp, 298–305. doi: 10.1016/j.vaccine.2021.11.065 PMC11705932 34895785

[B26] Jámbor C. Kozek-Langenecker S. A. Frietsch T. Knels R. Bux J. Sachs U. J.. (2008). Arbobacteria–pathogens transmittable by arthropods. Transfusion Med. Hemotherapy 35, 374–390. 10.1159/000112812 PMC3076330 21512627

[B27] Kamp H. D. Swanson K. A. Wei R. R. Dhal P. K. Dharanipragada R. Kern A.. (2020). Design of a broadly reactive Lyme disease vaccine. NPJ Vaccines 5, 33. doi: 10.1038/s41541-020-0183-8 32377398 PMC7195412

[B28] Klouwens M. J. Salverda M. L. M. Trentelman J. J. Ersoz J. I. Wagemakers A. Gerritzen M. J. H.. (2021). Vaccination with meningococcal outer membrane vesicles carrying Borrelia OspA protects against experimental Lyme borreliosis. Vaccine 39, 2561–2567. doi: 10.1016/j.vaccine.2021.03.059 33812741

[B29] Kumar M. Kaur S. Kariu T. Yang X. Bossis I. Anderson J. F.. (2011). *Borrelia burgdorferi* BBA52 is a potential target for transmission blocking Lyme disease vaccine. Vaccine. 29, 9012–9019. doi: 10.1016/j.vaccine.2011.09.035 21945261 PMC3202674

[B30] Lawrence R. S. Durch J. S. Stratton K. R. (Eds.) (2001). Vaccines for the 21st century: a tool for decisionmaking. (NationalAcademies Press). 25121214

[B31] Liekniņa I. Kozlova A. Šaško M. Akopjana I. Brangulis K. Tārs K. (2023). Evaluation of outer surface protein vaccine candidates of borrelia burgdorferi for lyme disease. Microbiol. Res. 14, 2022–2033. doi: 10.3390/microbiolres14040136

[B32] Livey I. O’Rourke M. Traweger A. Savidis-Dacho H. Crowe B. A. Barrett P. N.. (2011). A new approach to a Lyme disease vaccine. Clin. Infect. Dis. 52, s266–s270. doi: 10.1093/cid/ciq118 21217174

[B33] Lovrich S. D. Jobe D. A. Schell R. F. Callister S. M. (2005). Borreliacidal OspC antibodies specific for a highly conserved epitope are immunodominant in human Lyme disease and do not occur in mice or hamsters. Clin. Vaccine Immunol. 12, 746–751. doi: 10.1128/CDLI.12.6.746-751.2005 PMC1151971 15939749

[B34] Marcinkiewicz A. L. Lieknina I. Kotelovica S. Yang X. Kraiczy P. Pal U.. (2018). Eliminating factor H-binding activity of Borrelia burgdorferi CspZ combined with virus-like particle conjugation enhances its efficacy as a Lyme disease vaccine. Front. Immunol. 9, 181. doi: 10.3389/fimmu.2018.00181 29472926 PMC5809437

[B35] Nardelli D. T. Luedtke J. O. Munson E. L. Warner T. F. Callister S. M. Schell R. F. (2010). Significant differences between the Borrelia-infection and Borrelia-vaccination and-infection models of Lyme arthritis in C3H/HeN mice. FEMS Immunol. Med. Microbiol. 60, 78–89. doi: 10.1111/j.1574-695X.2010.00721.x 20662925

[B36] Nayak A. Schüler W. Seidel S. Gomez I. Meinke A. Comstedt P.. (2020). Broadly protective multivalent OspA vaccine against Lyme borreliosis, developed based on surface shaping of the C-terminal fragment. Infection Immun. 88, 10–1128. doi: 10.1128/IAI.00917-19 PMC7093141 31932330

[B37] Pine M. Arora G. Hart T. M. Bettini E. Gaudette B. T. Muramatsu H.. (2023). Development of an mRNA-lipid nanoparticle vaccine against Lyme disease. Mol. Ther. 31, 2702–2714. doi: 10.1016/j.ymthe.2023.07.022 37533256 PMC10492027

[B38] Plotkin S. A. (2011). Correcting a public health fiasco: the need for a new vaccine against Lyme disease. Clin. Infect. Dis. 52, s271–s275. doi: 10.1093/cid/ciq119 21217175

[B39] Seiler K. P. Vavrin Z. Eichwald E. Hibbs J. B. Jr. Weis J. J. (1995). Nitric oxide production during murine Lyme disease: lack of involvement in host resistance or pathology. Infection Immun. 63, 3886–3895. doi: 10.1128/iai.63.10.3886-3895.1995 PMC173547 7558296

[B40] Sigal L. H. Zahradnik J. M. Lavin P. Patella S. J. Bryant G. Haselby R.. (1998). A vaccine consisting of recombinant Borrelia burgdorferi outer-surface protein A to prevent Lyme disease. New Engl. J. Med. 339, 216–222. doi: 10.1056/NEJM199807233390402 9673299

[B41] Small C. M. Ajithdoss D. K. Rodrigues Hoffmann A. Mwangi W. Esteve-Gassent M. D. (2014). Immunization with a Borrelia burgdorferi BB0172-derived peptide protects mice against Lyme disease. PLoS One 9, e88245. doi: 10.1371/journal.pone.0088245 24505447 PMC3914939

[B42] Sonnesyn S. W. Manivel J. C. Johnson R. C. Goodman J. L. (1993). A Guinea pig model for Lyme disease. Infection Immun. 61, 4777–4784. doi: 10.1128/iai.61.11.4777-4784.1993 PMC281234 8406878

[B43] Steere A. C. Coburn J. Glickstein L. (2004). The emergence of Lyme disease. J. Clin. Invest. 113, 1093–1101. doi: 10.1172/JCI21681 15085185 PMC385417

[B44] Steere A. C. Grodzicki R. L. Kornblatt A. N. Craft J. E. Barbour A. G. Burgdorfer W.. (1983). The spirochetal etiology of Lyme disease. New Engl. J. Med. 308, 733–740. doi: 10.1056/NEJM198303313081301 6828118

[B45] Steere A. C. Sikand V. K. Meurice F. Parenti D. L. Fikrig E. Schoen R. T.. (1998). Vaccination against Lyme disease with recombinant Borrelia burgdorferi outer-surface lipoprotein A with adjuvant. New Engl. J. Med. 339, 209–215. doi: 10.1056/NEJM199807233390401 9673298

[B46] Vogel F. R. (2000). Improving vaccine performance with adjuvants. Clin. Infect. Dis. 30, S266–S270. doi: 10.1086/313883 10875797

[B47] Wormser G. P. Nadelman R. B. Dattwyler R. J. Dennis D. T. Shapiro E. D. Steere A. C.. (2000). Practice guidelines for the treatment of Lyme disease. Clin. Infect. Dis. 31, S1–S14. 10.1086/314053 10982743

[B48] Wressnigg N. Pöllabauer E. M. Aichinger G. Portsmouth D. Löw-Baselli A. Fritsch S.. (2013). Safety and immunogenicity of a novel multivalent OspA vaccine against Lyme borreliosis in healthy adults: a double-blind, randomised, dose-escalation phase 1/2 trial. Lancet Infect. Dis. 13, 680–689. doi: 10.1016/S1473-3099(13)70110-5 23665341

[B49] Zhao T. Cai Y. Jiang Y. He X. Wei Y. Yu Y.. (2023). Vaccine adjuvants: mechanisms and platforms. Signal transduction targeted Ther. 8, 283. doi: 10.1038/s41392-023-01557-7 PMC10356842 37468460

[B50] Zhong W. Stehle T. Museteanu C. Siebers A. Gern L. Kramer M.. (1997). Therapeutic passive vaccination against chronic Lyme disease in mice. Proc. Natl. Acad. Sci. 94, 12533–12538. doi: 10.1073/pnas.94.23.12533 9356484 PMC25028

[B51] Zumstein G. Fuchs R. Hofmann A. Preac-Mursic V. Soutschek E. Wilske B. (1992). Genetic polymorphism of the gene encoding the outer surface protein A (OspA) of Borrelia burgdorferi. Med. Microbiol. Immunol. 181, 57–70. doi: 10.1007/BF00189424 1406458

